# Save Forests Through Sustainable Papermaking: Repurposing Herbal Waste and Maple Leaves as Alternative Fibers

**DOI:** 10.3390/ma18040910

**Published:** 2025-02-19

**Authors:** Haradhan Kolya, Chun-Won Kang

**Affiliations:** Department of Housing Environmental Design, Research Institute of Human Ecology, College of Human Ecology, Jeonbuk National University, Jeonju 54896, Jeonbuk, Republic of Korea; hdk@jbnu.ac.kr

**Keywords:** sustainable papermaking, herbal waste, maple leaves, forest conservation, alternative cellulose fibers

## Abstract

This study explores a sustainable papermaking approach to contribute to forest conservation by repurposing delignified herbal waste and maple leaves as alternative cellulose sources. By reducing reliance on traditional wood-based materials, this method supports forest conservation while promoting environmental sustainability and creating economic opportunities from agricultural byproducts. Controlled experiments were conducted to extract cellulose and form paper using four fiber compositions: 100% leaf (P1), 100% herbal waste (P2), 75% leaf + 25% herbal waste (P3), and 75% leaf + 25% wood pulp (P4). Both treated and untreated herbal waste and leaves were characterized using Attenuated Total Reflection Fourier Transform Infrared Spectroscopy (ATR-FTIR) and X-ray Diffraction (XRD) to analyze chemical functionality and structural changes. The Kürschner cellulose content (22.4% in herbal waste and 15.2% in maple leaves) was determined through concentrated nitric acid and ethanol treatments, confirming high cellulose levels suitable for papermaking. Papers produced from these compositions exhibited enhanced mechanical properties, with the P2 sample (100% herbal waste) demonstrating the highest tensile strength (with P2 exhibiting a tensile strength of 1.84 kN/m) due to its elevated cellulose content. This innovative recycling approach contributes to deforestation reduction by valorizing agricultural waste materials, highlighting the feasibility of integrating alternative fibers into paper manufacturing. These findings present a promising pathway toward an eco-friendly, forest-saving paper industry while adding economic value to agro-waste resources.

## 1. Introduction

Paper has been a crucial material throughout human history, characterized by a layered structure of cellulose fibers interconnected through intermolecular forces such as van der Waals and hydrogen bonds [1,2]. These bonds are particularly vulnerable to moisture; as the water content of paper increases, the strength of these bonds diminishes, leading to a loss of structural integrity [3]. Traditionally, paper is produced by pressing moist fibers primarily derived from wood pulp into sheets that are then dried [4]. The origins of this process date back to around 105 A.D. in ancient China, where it is credited to Ts’ai Lun [5]. His method involved grinding the bark of shrubs and combining it with fibers from hemp, mulberry bark, and rags [6]. This mixture was processed with a stone mill, washed, and then spread onto a drain board to form a wet paper web, which was subsequently dried in the sun.

Despite its historical roots, the paper industry has continually evolved to meet the demands for higher quality, cost efficiency, and faster production [4]. The advent of information and communication technologies led to forecasts of a decline in paper consumption due to the rise in digital media [7]. Contrary to these predictions, the proliferation of computers and digital devices has actually increased the demand for paper, particularly for printing and writing purposes [8]. Additionally, the use of tissue papers (both dry and wet) and food packaging papers has increased tremendously due to modernization. As a result, global paper consumption has significantly risen, with substantial increases observed over recent decades [9].

Historically, the global paper industry has relied heavily on wood fibers for pulp production, with wood accounting for approximately 90–95% of all paper pulp [10]. Common sources include softwoods such as spruce, pine, and fir, as well as hardwoods like eucalyptus and birch [11]. However, the escalating demand for paper has led to a significant depletion of wood resources and accelerated deforestation [12]. This situation highlights the urgent need to explore alternative sources of cellulose to reduce dependence on wood..

Non-wood fibers, which now account for approximately 6.5% of global pulp production [11], have emerged as promising alternatives to wood-based sources [13]. In many developing countries, about 60% of cellulose fibers are derived from non-wood materials, such as bagasse (sugarcane fibers), cereal straw, bamboo, reeds, jute, flax, and banana stem [14,15,16,17]. These materials not only provide a renewable cellulose source but also promote a circular economy by repurposing agricultural waste, thus minimizing environmental impact. Countries like China and India lead this shift, sourcing over 70% of their pulp raw materials from non-wood fibers [18,19].

In recent years, herbal waste has gained attention as a non-wood fiber source, largely due to its high cellulose content and availability as a byproduct of the growing herbal and pharmaceutical industries [20]. The use of medicinal plants is highly diverse, with over 50,000 flowering plants known for their therapeutic properties [21]. Notable examples include *Aloe vera*, *Piper longum*, *Calendula officinalis*, *Camellia sinensis*, and *Clitoria ternatea*. The growing popularity of Ayurveda and organic products has driven an annual increase of approximately 12 million tons of herbal byproducts [22]. This large-scale production of herbal waste raises significant environmental concerns due to the limited strategies for its effective disposal or reuse [23,24,25].

Currently, residual materials from herbal medicine production are often discarded or repurposed for applications such as fertilizers [26], solid fuel [27], biochar [28], biogas [29], sound absorption [20], and sugar and amino acid extraction [30]. While these applications provide partial solutions, further exploration is needed to enhance the utility of herbal waste, particularly in areas like pulp and paper production. Addressing this gap could promote more sustainable waste management practices and contribute to the development of innovative, eco-friendly materials.

Similarly, sugar maple (*Acer saccharum*) leaves, known for their association with timber and maple syrup production [31], have also been recognized for their antibacterial properties [32] and ability to adsorb metal ions [33]. Recent studies indicate that chemically treated maple leaves show potential for use in sound absorption panels [34]. However, to maximize the utilization of maple leaves and prevent them from being discarded as waste, further exploration is needed, particularly in areas such as pulp and paper production.

Modern papermaking requires substantial water, energy, and chemical use, raising environmental concerns. Traditional chemical processes, like bleaching to remove lignin, use harmful agents that negatively impact human health and aquatic ecosystems [35,36]. Recent advancements in eco-friendly pulping processes, such as hydrogen peroxide and acetic acid treatments, have shown promise for lignin removal under mild reaction conditions, preserving cellulose while minimizing environmental impact [20,37]. Studies on agricultural residues and herbal waste suggest that these processes improve cellulose yield and enhance fiber crystallinity, making them well suited for sustainable pulp production.

This study aims to evaluate the potential of delignified herbal waste and maple leaves as alternative sources of cellulose for papermaking to contribute to forest conservation and sustainable economic development. By valorizing agricultural byproducts like herbal waste, this research supports environmental sustainability and contributes to a circular bioeconomy by transforming agro-waste into valuable products. These findings offer an innovative pathway toward forest-saving, sustainable papermaking, reducing reliance on wood fibers, and promoting environmental sustainability in the paper industry.

## 2. Materials and Methods

### 2.1. Materials

Acetic acid (>99%, CAS No: 64-19-7) and hydrogen peroxide (30%, CAS No: 7722-84-1) were obtained from Merck, Korea. Herbal waste was sourced from Itti Herb in Paju City, Gyeonggi-do, South Korea, and dried at room temperature (26 °C) for 20 days, reducing its moisture content from 19% to 9% (room dried). This waste included residues from various plants such as *Panax ginseng*, *Aspilia ovata*, *Wolfiporia extensa*, *Glycyrrhiza uralensis*, *Rubus coreanus*, *Angelica gigas*, *Paeonia japonica*, *Cnidium officinale*, *Astragalus membranaceus*, *Cinnamomum cassia*, and *Zingiber officinale* [20]. Additionally, sugar maple (*Acer saccharum*) leaves were collected from around Jeonbuk National University. The stems were removed, and the leaves were torn into small pieces. Balsa wood (*Ochroma pyramidale*) disk samples (29 mm in diameter and 10 mm thick), available in the laboratory, were also used in this study. Deionized water was used throughout the experiment.

### 2.2. Preparation of Pulp

**Figure 1:**
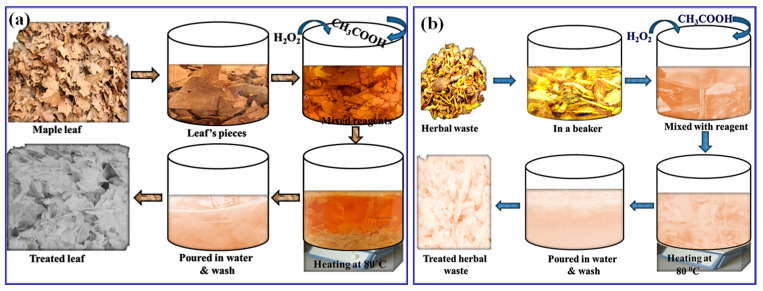
Schematic representation of the chemical treatment process applied to (**a**) maple leaves and (**b**) herbal waste.

This study chose specific treatment parameters, such as temperature, time, and reagent concentration, based on their effectiveness in maximizing cellulose extraction while minimizing fiber degradation. The selected conditions, an 85 °C treatment temperature, 80 min of reaction time, and a 1:1 mixture of glacial acetic acid and 30% hydrogen peroxide, were optimized through preliminary testing to ensure efficient delignification of herbal waste and maple leaves from our previous studies [20,34]. These parameters were chosen to balance lignin removal and cellulose preservation, which is crucial for maintaining the structural integrity and quality of the final pulp. The 85 °C temperature and 80 min reaction time were selected because they provide sufficient thermal energy for lignin breakdown without excessively high temperatures that could degrade cellulose and hemicellulose. The 1:1 acetic acid and hydrogen peroxide mixture was chosen as an environmentally friendly alternative to traditional pulping chemicals, as it selectively targets lignin and other extractives without excessive cellulose loss [38]. A schematic of the preparation process is shown in Figure 1.

### 2.3. Paper Making Process

**Figure 2:**
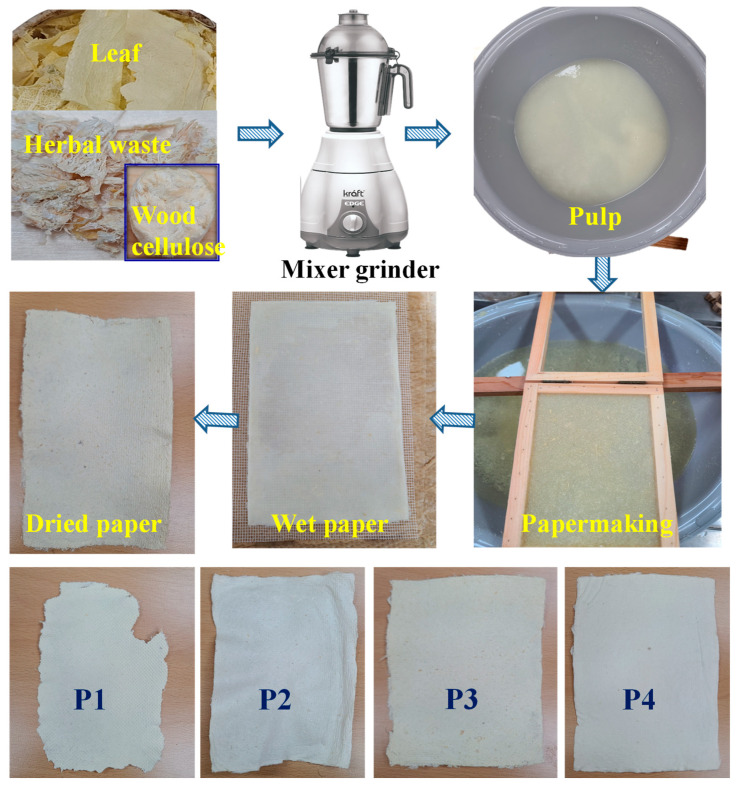
Detailed schematic of the papermaking process using dried and delignified leaves, herbal waste, and wood cellulose.

Dried and delignified leaves, herbal waste, and balsa wood cellulose were mixed in various weight percentages to produce pulp: 100% leaf (P1), 100% herbal waste (P2), 75% leaf + 25% herbal waste (P3), and 75% leaf + 25% balsa wood cellulose (P4). These ratios were randomly selected to provide an initial assessment of pulp and paper production using different combinations of leaves, herbal waste, and wood cellulose. The mixtures or individual cellulose samples were placed in a mixer grinder with sufficient water and processed for approximately 2 min to form pulp. The resulting pulp was then transferred to a large plastic container, where additional water was added and thoroughly mixed. Paper was formed using available equipment, as shown in Figure 2. Excess water was removed using a sponge and cloth. The prepared paper was then placed under a wooden plate with a 1 kg weight bar applied for pressure and dried in a hot air oven. A schematic of the papermaking process is shown in Figure 2. The selected fiber compositions were chosen to explore the individual and combined effects of non-wood fibers on paper properties. Each composition serves a distinct purpose: P1 and P2 provide a baseline for evaluating the mechanical and structural contributions of leaf and herbal waste fibers individually, while P3 examines the benefits of blending these non-wood fibers. P4, incorporating wood pulp, offers a comparison to traditional papermaking. This approach allows for a comprehensive assessment of the potential of herbal waste and maple leaves as sustainable alternatives, shedding light on how different fiber ratios can be optimized for diverse papermaking applications.

### 2.4. Kürschner Cellulose Analysis

The balsa wood, herbal waste, and leaves treated with hydrogen peroxide and acetic acid showed cellulose contents of 48.2%, 26.4%, and 17.1%, respectively. To compare the cellulose content before and after pulping, Kürschner cellulose analysis was conducted using a concentrated nitric acid and ethanol mixture (1:4 *v*/*v*). This process converts lignin into nitrated lignin, which dissolves in ethanol, leaving behind a white residue known as Kürschner cellulose [39,40,41,42]. The procedure involved treating 2 g of each dried, leaves, herbal waste, and balsa wood samples for 1 h. After extraction, the residue was vacuum filtered using a sintered glass crucible (Grade 4) and washed with distilled water and ethanol until the pH reached 7. The residue was then dried to a constant weight in a hot air oven at 105 °C. The cellulose content was calculated using the following Equation (1) [43].

(1) $$W=\frac{{(M}_{1}-M_{2})}{M_{0}}\times 100$$

where *W* represents the cellulose content (%), M_2_ is the weight of the dried crucible (g), M_1_ is the weight of the dried crucible with the residue (g), and M_0_ is the weight of the original sample (g). The prepared cellulose samples were stored in glass bottles for further characterization.

### 2.5. Characterization of Materials

#### 2.5.1. ATR-FTIR

Attenuated Total Reflection Fourier Transform Infrared Spectroscopy (ATR-FTIR) was used to assess changes in the functional groups of both untreated and treated herbal waste, leaf cells, and wood cell walls. The analysis was performed using a Perkin Elmer Frontier instrument (Waltham, MA, USA), with a scanning range from 400 cm^−1^ to 4500 cm^−1^. Additionally, the prepared cellulose samples were also scanned to identify any functional changes.

#### 2.5.2. XRD Analysis

An X-ray diffractometer (Xpert Pro) from PANalytical Multi-Purpose High-Performance (Almelo, The Netherlands) was utilized to study the crystallinity of the prepared Kürschner cellulose. The experimental setup included symmetrical reflection and transmission modes, with sample dimensions of 0.2 mm × 0.22 mm. The source was CuKα_1_ with a wavelength of 1.540 Å and CuKα_2_ with a wavelength of 1.544 Å. The divergence slit was fixed at 0.19 mm, with the generator set at 40 kV and a tube current of 30 mA. The scan axis was set to Gonio, covering a range from 5° to 60°, with a scan step size of 0.033°, totaling 1645 points in a continuous scan type, and a time per step of 50.16 s. The intensity was recorded automatically as a function of the scattering angle 2θ through a θ–2θ scan.

### 2.6. Characterization of Paper Sheets

#### 2.6.1. Basic and Mechanical Properties Analysis

The samples (P1–4) were sent to the Changgang Paper Technology Research Institute (CIPST) in Korea for evaluation of their basic characteristics and mechanical properties. The basic characteristics assessed included weight (g/m^2^), thickness (μm), smoothness (Benson method) (mL/min), and glossiness (%) of both surface and sides using an L&W Gloss Tester. The mechanical properties evaluated were tensile strength (kN/m) using an L&W Tensile Tester and elongation at break (%) using an L&W Bendtsen Tester (Kista, Sweden).

#### 2.6.2. Surface Analysis

A 3D Optical Profilometer (VR-6000) from Keyence (Osaka, Japan) was utilized to examine the surface roughness of paper sheets made from various compositions: 100% leaf (P1), 100% herbal waste (P2), 75% leaf + 25% herbal waste (P3), and 75% leaf + 25% wood waste (P4). This instrument offers high-resolution imaging and precise surface topography measurements, providing detailed data on parameters such as arithmetical mean height (S_a_), root mean square roughness (R_q_), average maximum height of the surface (S_z_), texture aspect ratio (S_tr_), the spatial parameter of the surface (S_pc_), and developed interfacial area ratio (S_dr_) [44]. Together, these parameters offer a thorough characterization of the surface texture. Additionally, surface characteristics were analyzed using a Scanning Electron Microscope (SEM), model Genesis-1000 from Emcrafts, Seoul, Korea. The samples, approximately 1 cm^2^ in size, were coated with gold ions using an ion sputter coater, model SCM from Emcrafts, Seoul, Korea, prior to SEM analysis.

#### 2.6.3. Color Analysis

A spectrophotometer (Konica Minolta CR-400, Tokyo, Japan) was used to examine the color of each sample. This instrument quickly measures the color of various substrate surfaces under a D65 light source, assessing lightness and chromatic properties. The color difference (∆E*) was calculated using the CIELAB color system, based on the chromatic coordinates L* (lightness), a* (red–green axis), and b* (yellow–blue axis), as expressed in Equation (2).

(2) $${∆E}^{*}=\sqrt{({L^{*}}_{2}-{L^{*}}_{1}{)}^{2}+({a^{*}}_{2}-{a^{*}}_{1}{)}^{2}+({b^{*}}_{2}-{b^{*}}_{1}{)}^{2}}$$

where L* represents the lightness of the sample surface, a* corresponds to the red–green chromatic coordinate, and b* corresponds to the yellow–blue chromatic coordinate.

#### 2.6.4. Moisture Absorption Test

We have conducted the moisture absorption test using a gravimetric method, where the samples (size 55 × 52 mm) were exposed to a controlled humidity environment (60%, at 25 °C) and their weight change was recorded after 24 h.

## 3. Results and Discussions

### 3.1. Characterization of Materials

**Figure 3:**
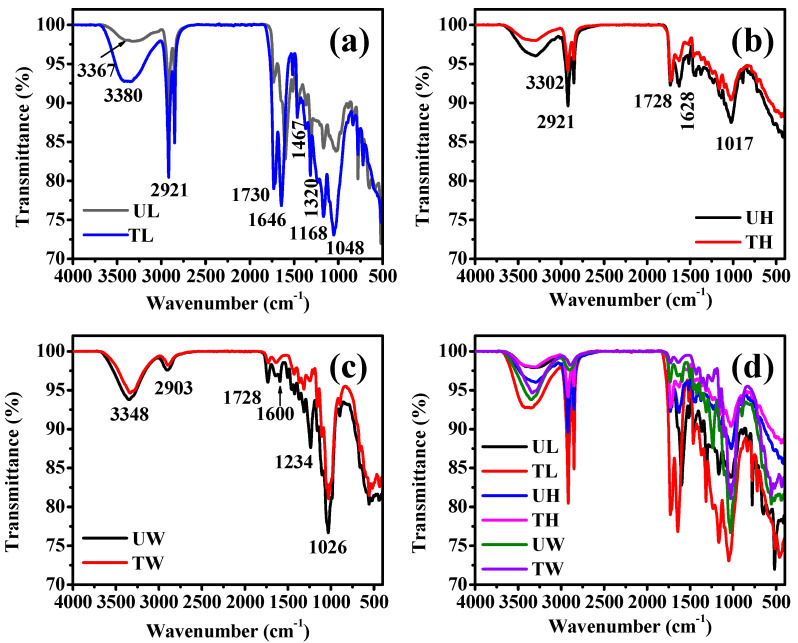
IR spectra of (**a**) untreated leaf (UL), treated leaf (TL), (**b**) untreated herbal waste (UH), treated herbal waste (TH), (**c**) untreated balsa wood (UW), treated balsa wood (TW), and (**d**) comparative IR spectra of untreated and treated samples of leaf, herbal waste, and balsa wood.

The ATR-FTIR spectra analysis (Figure 3) reveals notable differences between untreated and treated samples of leaf, herbal waste, and balsa wood. Untreated leaf (UL) exhibits peaks at 3367, 2921, 1597, 1467, 1320, and 1021 cm^−1^ (Figure 3a), indicating the presence of O-H, C-H, aromatic rings, and C-O groups [34].

In contrast, treated leaf (TL) shows a high-intensity peak at 3380 cm^−1^ and additional peaks at 1730, 1646, 1168, and 1048 cm^−1^, suggesting increased O-H groups and structural changes in cellulose [34]. Untreated herbal waste (UH) has peaks at 3302, 2921, 1728, 1628, and 1017 cm^−1^ (Figure 3b), which indicate the presence of O-H, C-H, C=O, and C-O functional groups. The peak at 1728 cm^−1^ is characteristic of carbonyl groups, likely from lignin and other organic components in the herbal waste, while treated herbal waste (TH) shows reduced intensity at these peaks, indicating lignin removal. The reduction in peak intensity compared to UL suggests the extraction of herbal chemicals and lignin removal due to hydrogen peroxide and acetic acid treatment, resulting in a less complex and more purified cellulose structure. Untreated balsa wood (UW) shows peaks at 3348, 2903, 1728, 1600, 1234, and 1026 cm^−1^ (Figure 3c), with treated balsa wood (TW) displaying lower intensity at these positions, signifying effective lignin extraction [38,45]. Comparative IR spectra of all the above samples are shown in Figure 3d. The treatments using hydrogen peroxide and acetic acid effectively remove lignin and other non-cellulosic materials, resulting in a more purified cellulose structure in the treated samples.

**Figure 4:**
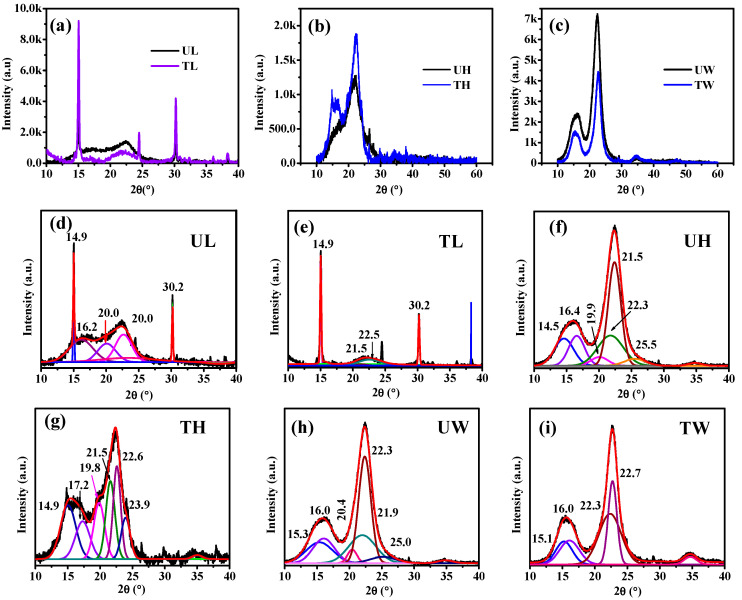
XRD spectra of (**a**) leaves, (**b**) herbal waste, and (**c**) balsa wood. Deconvoluted spectra of (**d**) untreated leaves (UL), (**e**) treated leaves (TL), (**f**) untreated herbal waste (UH), (**g**) treated herbal waste (TH), (**h**) untreated wood (UW), and (**i**) treated wood (TW).

The XRD analysis of untreated and treated samples of leaves, herbal waste, and balsa wood revealed notable changes in crystallinity, which can be correlated with the structure of cellulose Iβ (monoclinic) (Figure 4a–c). This suggests that the treatment process effectively enhanced its crystalline structure by removing amorphous components such as lignin and hemicellulose. The deconvoluted XRD spectra provide further insight into these findings (Figure 4d–i). In untreated leaves (UL), peaks were observed at 2θ values of 14.9°, 16.2°, 20.0°, 22.6°, and 30.2°, corresponding to the Miller indices (1−10), (110), and (020) of cellulose Iβ, while the peak at 21.5°and 21.9° was attributed to the amorphous regions within the cellulose structure [38,46,47]. After treatment, the leaves (TL) exhibited fewer peaks, notably at 14.9°, 21.5°, 22.5°, and 30.2°, indicating a reduction in amorphous material and a more ordered crystalline structure (Figure 4e).

For herbal waste, the untreated sample (UH) displayed peaks at 14.5°, 16.4°, 19.9°, 21.5°, 22.3°, and 25.5°, with peaks at 14.5°, 16.4°, and 22.3° corresponding to the Miller indices of cellulose Iβ (Figure 4f). The treated sample (TH) showed peaks at 14.9°, 17.2°, 19.8°, 21.5°, 22.6°, and 23.9°, reflecting an enhanced crystalline structure post-treatment (Figure 4g). In the case of balsa wood, the untreated sample (UW) exhibited peaks at 15.3°, 16.0°, 20.4°, 21.9°, 22.3°, and 25.0° (Figure 4h). In comparison, the treated sample (TW) showed fewer peaks at 15.1°, 16.0°, 22.3°, and 22.7°, indicating a change in crystallinity likely due to partial degradation during treatment (Figure 4i) [38].

### 3.2. Kürschner Cellulose Analysis

The cellulose content in leaf, herbal waste, and balsa wood samples treated with acetic acid and hydrogen peroxide was 17.1%, 26.4%, and 48.2%, respectively. However, Kürschner cellulose analysis revealed that maple leaves contained 15.2% cellulose, indicating a considerable cellulose concentration after removing other components. Similarly, herbal waste showed a cellulose content of 22.4%. In contrast, balsa wood exhibited a high % cellulose content of 42.4%. It indicates approximately 10% less cellulose than the acetic acid and hydrogen peroxide-treated sample. This reduction may be attributed to cellulose degradation or losses during filtration and washing. The herbal waste samples could contain different amounts of cellulose due to the variable mixture of herbal components present in the samples.

### 3.3. Characterization of Paper Sheets

#### 3.3.1. Basic and Mechanical Properties Analyses

The evaluation of the basic and mechanical properties of the paper samples P1, P2, P3, and P4 reveals notable insights into the performance and quality of each composition. Table 1 summarizes the results of these evaluations.

The analysis of the basic and mechanical properties of the paper samples reveals differences influenced by their fiber compositions [48]. The sample composed of 75% leaf and 25% herbal waste (P3) exhibited the highest weight (107.8 g/m^2^) and thickness (410 μm), reflecting a denser and more compact structure compared to the other samples. This increased density can be attributed to the synergistic interaction between leaf and herbal waste fibers [49], resulting in enhanced fiber entanglement and reduced porosity. In contrast, the sample containing 75% leaf and 25% wood waste (P4) was the lightest and thinnest (59.4 g/m^2^ and 222 μm), suggesting a less compact structure, likely due to the smoother, less entangled nature of the wood fibers. Despite differences in composition, all samples exhibited a uniform smoothness value of 3480 mL/min. However, this consistency is likely attributable to the saturation limit of the instrument, indicating that the surfaces are too rough for accurate measurement using the current method. Glossiness varied notably across the samples, with P4 displaying the highest gloss values (3.6% on the surface and 3.8% on the sides). This can be attributed to the inclusion of wood cellulose, known for producing smoother and more reflective surfaces [50]. In contrast, the samples with higher herbal waste content exhibited lower glossiness, reflecting the rougher texture of non-wood fibers. Higher crystallinity typically indicates a more orderly arrangement of cellulose fibrils, which improves tensile strength and stiffness through enhanced intermolecular bonding. Conversely, P4, which incorporated wood waste, showed the lowest elongation at break (0.5%), reflecting the rigid and less elastic nature of wood cellulose. However, P4 maintained a balance of tensile strength and glossiness, making it suitable for applications where surface quality is prioritized.

These findings highlight the potential of using both leaf and herbal waste as sustainable alternatives to traditional wood pulp, offering a combination of good mechanical strength and flexibility. The results also emphasize the versatility of blending different fiber compositions to achieve some paper properties.

#### 3.3.2. Surface Analyses

**Figure 5:**
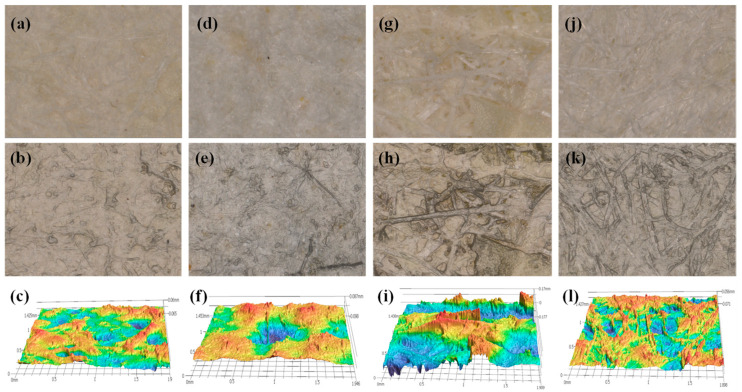
Optical, 2D, and 3D images of samples: (**a**–**c**) P1, (**d**–**f**) P2, (**g**–**i**) P3, and (**j**–**l**) P4 [100% leaf (P1), 100% herbal waste (P2), 75% leaf + 25% herbal waste (P3), and 75% leaf + 25% wood waste (P4)].

The surface color differences were quantified using the CIELAB color system, Chroma Meter CR-400 (Tokyo, Japan). Specifically, the ΔE* values were measured relative to the P1 samples, with ΔE* = 4.06 ± 0.46 when comparing P1 to P2, ΔE* = 3.45 ± 0.97 for P1 to P3, and ΔE* = 4.53 ± 0.34 for P1 to P4. These findings indicate that there are noticeable differences in the color properties among the samples, suggesting that variations in fiber composition influence the overall appearance of the paper. In addition, the optical and 3D images of the surfaces are presented in Figure 5, while Table 2 summarizes the roughness parameters: arithmetical mean height (S_a_), root mean square roughness (S_q_), average maximum height of the surface (S_z_), texture aspect ratio (S_tr_), spatial parameter of the surface (S_pc_), and developed interfacial area ratio (S_dr_). These parameters provide a comprehensive understanding of the surface texture. Figure 5a–c show the optical, 2D, and 3D images of sample P1, while Figure 5d–f, Figure 5g–i, and Figure 5j–l represent the corresponding images for samples P2, P3, and P4, respectively. These images illustrate the fiber distribution across the paper sheets, providing insights into surface characteristics and texture variations among the different compositions.

The surface roughness analysis of paper samples P1, P2, P3, and P4, conducted using a 3D profilometer at 160× magnification, revealed differences in surface characteristics influenced by fiber composition. Sample P3, composed of 75% leaf and 25% herbal waste, exhibited the highest surface roughness, with an arithmetical mean height (S_a_) of 28.656 μm, root mean square roughness (S_q_) of 36.478 μm, and an average maximum height (S_z_) of 195.353 μm. This indicates a notably rough and textured surface, likely resulting from the fibrous interaction between leaf and herbal waste materials. In comparison, the 100% herbal waste sample (P2) and the 75% leaf + 25% wood waste sample (P4) demonstrated moderate roughness, with S_a_ values of 17.348 μm and 14.742 μm, respectively. The 100% leaf sample (P1) displayed the smoothest surface, characterized by the lowest S_a_ (12.017 μm) and S_q_ (15.468 μm).

The texture aspect ratio (S_tr_) analysis revealed that P3 had the least isotropic texture, indicating greater surface variability, while P2 and P4 exhibited more uniform, isotropic surfaces. Additional parameters, such as the spatial parameter (S_pc_) and developed interfacial area ratio (S_dr_), confirmed that P3 had the most irregular and complex surface topography, further reinforcing the impact of the herbal waste–leaf fiber combination. These results suggest that the integration of herbal and wood waste affects surface texture, with the P3 composition yielding the roughest surface profile.

The findings between mechanical properties and surface roughness, as presented in Table 1 and Table 2, highlights the influence of fiber composition on paper performance. For example, P3 (75% leaf + 25% herbal waste) exhibited the highest weight and thickness, followed by P2 (100% herbal waste), P1 (100% leaf), and P4 (75% leaf + 25% wood waste), indicating that the addition of herbal waste enhances density and thickness. The highest tensile strength and elongation at break were observed in P2, demonstrating that herbal waste fibers not only contribute to surface roughness but also reinforce mechanical strength and flexibility. The high roughness in P3 correlates with its denser, more fibrous structure, reflecting the synergistic effects of combining leaf and herbal waste fibers.

Notably, the enhanced mechanical performance of P2 aligns with previous studies, where panels made from bagasse fibers exhibited superior physical and mechanical properties compared to panels made from poplar and mixed hardwoods. Similarly to bagasse, herbal waste fibers contribute to increased surface roughness and improved structural integrity [51]. In contrast, the smoother surfaces of P1 and P4 make them more suitable for applications requiring finer finishes, with P4 showing the highest glossiness, attributable to its lower roughness and wood cellulose content. These findings reinforce the potential of herbal and leaf waste as viable alternatives to wood cellulose in papermaking, offering a balance between mechanical performance and surface characteristics depending on fiber composition.

The moisture absorption test was conducted by comparing the initial and final weights of each sample and calculating the percentage increase. The results showed that sample P1 absorbed approximately 0.62% moisture, P2 about 0.71%, P3 only 0.11%, and P4 approximately 0.89%. These differences suggest that the fiber composition and the resulting paper structure influence moisture uptake.

**Figure 6:**
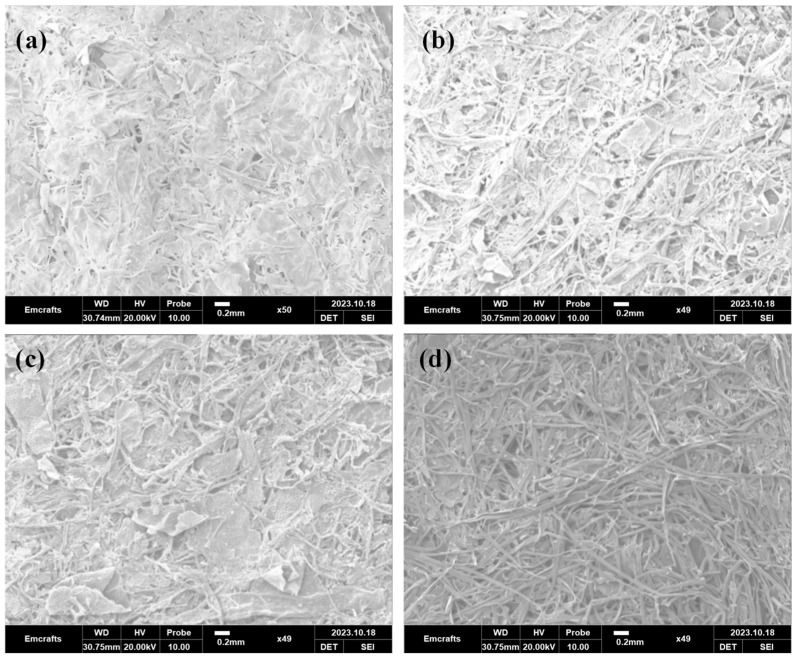
SEM images of the samples: (**a**) P1, (**b**) P2, (**c**) P3, and (**d**) P4 [100% leaf (P1), 100% herbal waste (P2), 75% leaf + 25% herbal waste (P3), and 75% leaf + 25% wood waste (P4)].

Additionally, SEM analysis was conducted to visualize the fibrous properties more clearly. The SEM images, shown in Figure 6, along with the optical images, support the isotropic properties of the P2 and P4 samples and provide insights into the surface roughness. Furthermore, using ImageJ software (online, v.1.50d), the average fiber diameters were quantified as follows: P1: 0.031 ± 0.008 mm, P2: 0.052 ± 0.017 mm, P3: 0.040 ± 0.006 mm, and P4: 0.047 ± 0.008 mm.

## 4. Concept of Practical Utility

The existing literature indicates that producing one ton of paper typically requires approximately 24 trees, each about 40 feet tall and 6–8 inches in diameter [52]. In contrast, non-wood fibers such as agricultural residues, bamboo, and herbal waste have been shown to be viable alternatives, with the raw material requirement varying considerably based on cellulose content and fiber quality. For instance, bamboo fibrils have been reported to contain cellulose at a volumetric percentage of around 73.83% [10], and studies on bagasse fibers have demonstrated cellulose contents ranging from 40% to 45%, with bagasse-based paper exhibiting mechanical properties comparable to those of conventional wood-based paper [53]. In our study, initially findings indicate that delignified herbal waste and maple leaves contain 22.4% and 15.2% cellulose, respectively, which supports their potential as viable raw materials. Additionally, the mechanical properties of the resulting paper, particularly the enhanced tensile strength and flexibility observed in herbal waste-based samples, align with the performance characteristics reported for other non-wood fiber-based papers [54]. Although the cellulose content of herbal waste and maple leaves is lower than that of bamboo or bagasse, their incorporation into paper production may still offer important environmental benefits. The enhanced tensile strength and flexibility observed in herbal waste-based samples could be useful to the industrial viability of these materials for producing paper products, including tissues, packaging, and stationery. This approach could provide a sustainable solution for enhancing the utilization of plant-based waste, contributing to reducing deforestation and minimizing the environmental footprint of conventional papermaking processes. By utilizing abundant, non-wood fibers, manufacturers can lower wood consumption, cut production costs, and respond to the increasing global demand for paper more sustainably and resource-efficiently.

This study lacks precise data on the substitution rate of herbal waste and maple leaves for large-scale paper production. While initial results are promising, further optimization of fiber ratios and pilot trials are needed. Long-term durability testing and economic feasibility assessments are also required. Future research will focus on refining the process and scaling up production.

## 5. Conclusions

This study underscores the potential of delignified herbal waste and maple leaves as sustainable raw materials for papermaking. With cellulose content of 22.4% in herbal waste and 15.2% in maple leaves, the resulting paper sheets revealed mechanical properties suitable for packaging, tissue, and stationery applications. ATR-FTIR and XRD analyses confirmed effective lignin removal and cellulose preservation.

By repurposing agro-waste, this research helps circular economy practices, reduces wood reliance, and lowers environmental impact. While further optimization is needed for broader industrial use, these findings present a sustainable pathway for enhancing sustainability and resource efficiency in the paper industry.

## Figures and Tables

**Table 1:** Basic and mechanical properties of paper samples made from different fiber compositions: 100% leaf (P1), 100% herbal waste (P2), 75% leaf + 25% herbal waste (P3), and 75% leaf + 25% wood waste (P4).

Samples		Weight (g/m^2^)	Thick (μm)	Smoothness (mL/min)	Glossiness		Tensile Strength (kN/m)	Elongation at Break (%)
					Surface (%)	Sides (%)		
P1	Ave.	72.4	366	3480	2.4	1.3	0.31	0.60
	Stdv.	6.2	70	0	0.2	0.3	0.15	0.23
P2	Ave.	86.6	299	3480	2.9	2.7	1.84	0.80
	Stdv.	3.7	36	0	0.4	0.5	0.25	0.13
P3	Ave.	107.8	410	3480	2.4	2.6	0.27	0.60
	Stdv.	8.0	13	0	0.2	0.2	0.07	0.21
P4	Ave.	59.4	222	3480	3.6	3.8	0.35	0.5
	Stdv.	0	40	0	0.2	0.3	0.02	0.09

**Table 2:** Surface roughness parameters of paper samples P1, P2, P3, and P4 measured using a 3D profilometer at 160x magnification [100% leaf (P1), 100% herbal waste (P2), 75% leaf + 25% herbal waste (P3), and 75% leaf + 25% wood waste (P4)].

Samples		S_a_ (μm)	S_z_ (μm)	S_tr_	S_pc_ (1/mm)	S_dr_	Sq(μm)
P1	Ave.	12.017	99.742	0.475	110.083	0.198	15.468
	Stdv.	1.517	10.017	0.205	4.146	0.054	2.297
P2	Ave.	17.348	116.775	0.688	145.975	0.210	20.698
	Stdv.	6.774	17.024	0.043	60.045	0.070	6.635
P3	Ave.	28.656	195.353	0.316	214.012	1.002	36.478
	Stdv.	5.523	37.965	0.164	56.956	0.494	5.84
P4	Ave.	14.742	101.365	0.624	187.582	0.487	17.684
	Stdv.	2.075	7.379	0.066	18.612	0.106	2.025

## Data Availability

The original contributions presented in this study are included in the article. Further inquiries can be directed to the corresponding author.
